# Microenvironmental and Molecular Pathways Driving Dormancy Escape in Bone Metastases

**DOI:** 10.3390/ijms262411893

**Published:** 2025-12-10

**Authors:** Mohamad Bakir, Alhomam Dabaliz, Ahmad Dawalibi, Khalid S. Mohammad

**Affiliations:** 1 Department of Medicine, College of Medicine, Alfaisal University, Riyadh 11533, Saudi Arabia; mbakir@alfaisal.edu; 2 Department of Clinical Skills, College of Medicine, Alfaisal University, Riyadh 11533, Saudi Arabia; almdabaliz@alfaisal.edu; 3 Department of Biomedical and Translational Sciences Division, Sanford School of Medicine, University of South Dakota, Vermillion, SD 57069, USA; ahmad.dawalibi@coyotes.usd.edu; 4 Department of Anatomy, College of Medicine, Alfaisal University, Riyadh 11533, Saudi Arabia

**Keywords:** bone metastases, tumor dormancy, dormancy escape, bone marrow niche, neutrophil extracellular trap, TGF-β signaling

## Abstract

Bone metastases remain a leading cause of morbidity and mortality in patients with advanced breast, prostate, and lung cancers. A striking clinical feature of bone metastasis is the ability of disseminated tumor cells (DTCs) to persist in a dormant state for years or even decades before reawakening to drive overt disease. While the molecular and microenvironmental cues that induce and maintain dormancy have been increasingly studied, the mechanisms governing dormancy escape remain poorly defined yet are critical for preventing relapse. In this review, we synthesize emerging evidence on how the bone microenvironment orchestrates the transition of dormant tumor cells into proliferative lesions. We discuss how osteoclast-mediated bone resorption liberates growth factors such as TGF-β and IGF-1, fueling reactivation; how loss of osteoblast-mediated quiescence signals disrupts the endosteal niche; and how bone marrow adipocytes provide metabolic support through lipid transfer and adipokine secretion. We highlight the role of immune surveillance in maintaining dormancy and how immunosuppressive myeloid populations, regulatory T cells, and inflammatory triggers, such as neutrophil extracellular traps, promote escape. Additional emphasis is placed on extracellular matrix remodeling, mechanotransduction, angiogenic switching, and systemic factors, including aging, hormonal changes, and sympathetic nervous system activation. We also review epigenetic and metabolic reprogramming events within dormant cells that enable reactivation. Finally, we evaluate therapeutic strategies to sustain dormancy or prevent reawakening, including osteoclast-targeted therapies, immune-modulating approaches, and epigenetic or metabolic interventions. By integrating these insights, we identify key knowledge gaps and propose future directions to intercept dormancy escape and delay or prevent metastatic relapse in bone.

## 1. Introduction

The major cause of mortality in cancer is its ability to metastasize around the body, leaving the site of the primary tumor and spreading to different parts of the body [1]. Thus, in cancers that preferentially metastasize to the bones, like breast cancer, prostate cancer, and multiple myeloma, bone metastasis is a major contributor to their lethality burden [2]. However, not all cancers that spread develop to become lethal metastatic lesions, as a large amount of these malignant cells enter a state of dormancy upon reaching the bone, remaining quiet, hidden, and nonlethal for what may be many years [3]. While some studies have estimated that the majority of skeletal relapses due to reactivation of dormant tumor cells (DTCs) happen within 5 years of initial diagnosis [4], other studies have attributed skeletal relapses that occurred up to 20 years after initial diagnosis to this phenomenon [5].

The process of cancer dormancy has captured the attention of many researchers. Much has been discovered and understood about the processes required for DTCs to enter a dormancy state; however, the triggers for their escape from that state remain vaguer. Thus, this review aims to examine molecular, cellular, and systemic drivers of dormancy escape in bone, identify therapeutic vulnerabilities, and highlight open gaps within the literature.

The bone microenvironment is considered a very attractive niche for DTCs. This dynamic tissue plays a critical role in supporting these cells via the intricate interplay between the resident cells [6]. The continuous bone remodeling commandeered by osteoblasts and osteoclasts releases a variety of growth factors like TGF-β and IGF-1 into the environment that promote survival of cancer cells [6]. The rich immune system presence enforces the continuation of the dormant state of DTCs [7]. The bone marrow adipocytes (BMAs) assist DTC survival by providing them with free fatty acids for energy metabolism as well as adipokines that promote their survival [8,9]. The vascular environment within this niche promotes a state of stability and quiescence through stable nutrient delivery and secretion of certain factors [10]. Even the mechanical stress that bones go through due to locomotion causes unique changes within the bone microenvironment that may tip DTCs towards reactivation [11].

From the previous features that set the bone nice apart from other niches around the body, researchers have found targets for their studies to explore the main biological drivers of escape. Changes in bone remodeling, metabolic reprogramming of DTCs and bone-resident cells, loss of immune control, inflammatory events, ECM stiffening, promotion of angiogenesis, and different systemic changes like aging, hormonal alterations, and even stress have all been studied as possible drivers of DTC escape [12,13,14,15,16].

This exploration of DTCs and their biologic drivers has led to the conclusion that these cells exhibit a significant level of plasticity. DTCs seem to be able to read cues within the environment around them that push them to reactivate and re-enter the cell cycle. This ability appears to be secondary to epigenetic control like overexpression of certain tyrosine kinases, upregulation of Notch signaling, and deregulating the activity of histone lysine demethylase [17]. Another factor that seems to contribute to this feature in DTCs is their metabolic flexibility, such as their ability to switch between the use of anaerobic glycolysis to oxidative phosphorylation for their energy needs based on their environment [18].

As DTCs do not exhibit any lethal risk as long as they remain within their state of quiescence, it is clear that intercepting the process of reactivation and triggers of dormancy escape for these cells is the crucial therapeutic target for this process. Thus, this review will discuss the conceptual framework required for understanding this process before delving into the niche-driven mechanisms of escape as well as the molecular signaling pathways implicated in this process, and finally discussing the clinical and therapeutic implications of dormancy escape.

Breast cancer, particularly estrogen receptor-positive (ER+) variants, often enters an extended dormant phase in bone, with recurrences typically manifesting 8–10 years or even decades post initial diagnosis. Reactivation could happen after several years, leading to delayed metastatic relapse. (DTCs) are frequently located in bone marrow, and their existence is a significant indicator of late recurrence [3,12,19,20,21,22]. Prostate cancer is distinguished by its capacity to remain latent in bone for years or decades, with late recurrences being relatively representative of this cancer type. Similarly to breast cancer, prostate cancer (DTCs) can reemerge after prolonged dormancy, resulting in bone metastases [12,21]. Lung cancer generally demonstrates a significantly shortened latency period in bone, with dormancy lasting for merely 1–3 years, and the majority of recurrences happening within a few years post diagnosis. The aggressive characteristics of lung cancer result in fast dissemination and early metastatic proliferation, rendering long-term dormancy in bone less prevalent than in breast and prostate cancers [12].

## 2. Conceptual Framework: Dormancy vs. Escape

### 2.1. Definitions

To explain and explore cancer dormancy, two main models have been proposed: the first model, known as the “Tumor Mass Dormancy” model, posits that dormant DTCs are constantly proliferating and dying at a similar rate resulting in an equilibrium where the total mass of the tumor remains relatively unchanged [23]. This model relies on two mechanisms to explain this state: the first mechanism is angiogenic dormancy, which suggests that dormant DTCs induce angiogenesis to achieve this equilibrium, without tipping the scale towards excessive angiogenesis that would push the DTCs into a progressive phase. Thus, the dormant DTCs ensure a sufficient amount of nutrients to proliferate and replace the dying DTCs that are further away from the natural blood vessels [23,24]. The second mechanism is immunomediated dormancy, which refers to the role of immune cells, especially CD8+ T-cells, in maintaining dormancy of DTCs. This effect has been shown in animal studies where suppression using anti-CD8 significantly promoted growth and proliferation of DTCs [23,25].

On the other hand, the “Cellular Dormancy” model proposes that dormant DTCs are characterized by three features: minimum proliferation, minimum death, and reversibility [23]. Thus, this model contrasts with the tumor mass dormancy model as it suggests that dormant DTCs are in a state of non-proliferative quiescence. This model is more consistent with the classical view on tumor dormancy [26].

### 2.2. Key Regulators of Dormancy Maintenance

Many regulators for tumor dormancy have been described. Studies have described p38MAPK’s role in this process, as the balance between it and ERK in the extracellular matrix is a major molecular switch for dormancy [24,27]. Breaking this balance by suppressing p38MAPK by fibronectin and activating ERK by urokinase-plasminogen activator receptor (uPAR) has been shown to extract tumor cells from their dormancy and resume proliferative activity [28]. Tumor growth factor beta 2 (TGF-β2) is another factor that is associated with tumor dormancy [24]. In hypoxic conditions, this factor is upregulated in the bone marrow alongside proliferation inhibitor p27; both work together to maintain the state of dormancy in DTCs. In contrast, metastatic outgrowth was induced when TGF-β2′s levels were reduced [21,29]. The bone morphogenetic protein (BMP) family has shown importance in DTC dormancy as well, as BMP-7′s signaling through BMPR2 has been reported to maintain dormancy in DTCs in vitro and increase metastasis free survival in xenograft animal models [21]. Direct interactions between DTCs and osteoblast-lineage cells is another mechanism that promotes tumor dormancy. This pathway mainly relies on TAM receptor signaling. TAM receptors are located on DTCs and include AXL, TYRO, and MERTK. Osteoblasts interact with DTCs to induce AXL expression on them, then they bind to AXL via growth-arrest-specific 6 (GAS6) which signals DTCs to reduce proliferation and enter a state of dormancy [30]. GAS6/AXL interaction is a major controller of p38MAPK/ERK balance as well as TGF-β2 levels in the bone microenvironment, solidifying it as a central pathway for dormancy control in bones [30].

### 2.3. Transition to Escape

**Figure 1:**
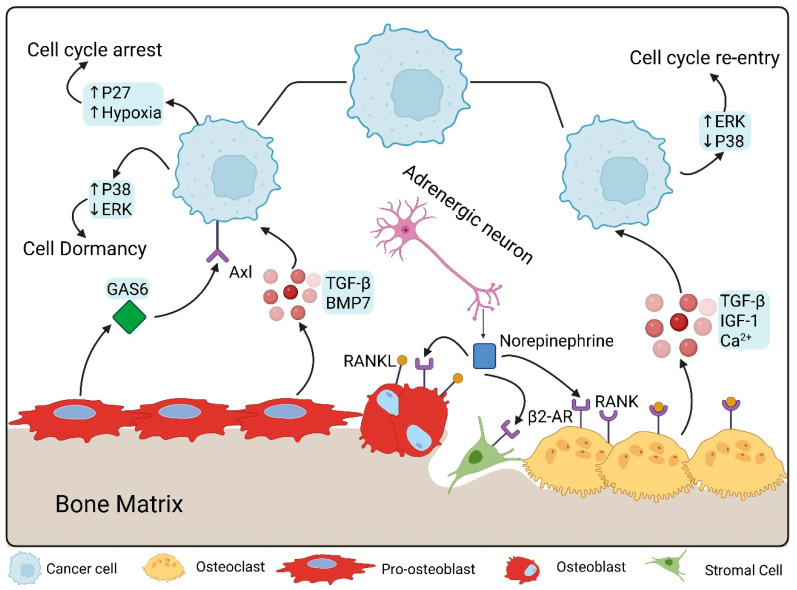
Schematic comparing opposing cues in the bone microenvironment that (**left**) maintain the dormancy of disseminated tumor cells (DTCs) versus (**right**) promote cell-cycle re-entry and tumor growth. On the dormancy side, interactions with osteoblast-lineage cells deliver GAS6–AXL signaling that shifts the intracellular balance toward p38 high/ERK low, stabilizes p27, and supports quiescence under hypoxic, endosteal conditions; BMP-7 and TGF-β2 further strengthen dormancy maintenance through p27 induction and ERK inhibition. On the activation side, osteoclast-mediated bone resorption releases TGF-β1, IGF-1, and Ca^2+^ from the matrix, tipping the balance toward ERK high/p38 low and encouraging cell-cycle re-entry; increased RANKL–RANK signaling amplifies osteoclastogenesis, reinforcing the resorptive feedback loop. Sympathetic/adrenergic input (norepinephrine→β2-adrenergic receptors on osteolineage/stromal cells) raises RANKL and pro-inflammatory mediators, further supporting tumor escape. These niche programs collectively illustrate a reversible switch between a dormancy-favorable endosteal state and a reactivation state driven by resumption and inflammation. Created in BioRender. Mohammad, K. (2025) https://BioRender.com/1i2pwh0 (accessed on 30 October 2025).

Disruptions in any of the key regulators mentioned previously may encourage dormant DTCs to escape shift to a proliferative phenotype. For example, while AXL maintains dormancy, GAS6′s interaction with TYRO and MERTK has been shown to promote dormancy escape [21]. Neuronal signaling with norepinephrine via β2-adrenergic receptors was also found to promote cancer cell proliferation in vitro [31]. In contrast with osteoblasts’ role to maintain dormancy, osteoclasts and bone resorption tip the scale more towards DTC proliferation and dormancy escape (Figure 1). Treatments that increase bone turnover have been shown to exacerbate this effect, while those that downregulate osteoclastic activity show reduced skeletal metastasis [30]. The exact mechanism behind this relationship between bone resorption and dormancy escape is not understood completely, and further studies are needed to explore this area [30] (Table 1).

## 3. Bone Microenvironment Cues Driving Dormancy Escape

### 3.1. Osteoclast Activity and Bone Resorption

As previously mentioned, osteoclasts play a role in dormancy escape for DTCs. The activity of osteoclast in the bone microenvironment results in bone absorption, which in turn releases many factors into the microenvironment like TGF-β1, insulin-like growth factor 1 (IGF-1), and calcium [32]. These factors in turn promote the proliferative activity in dormant DTCs via different mechanisms, pushing these DTCs to escape dormancy and enter into a proliferative phase [33,34]. Thus, osteoclast activation has been linked to DTC reactivation and escape from dormancy, which was seen in animal models, where treatment with receptor activator of nuclear factor κB ligand (RANKL) resulted in reactivation of dormant DTCs within the bone and triggered metastatic growth [35].

### 3.2. Osteoblast and Endosteal Niche Remodeling

DTCs seem to prefer localizing to the endosteal niche within the bone marrow [36]. This niche within the endosteum is located at the inner surface of the bone cavity, and primarily houses undifferentiated osteoblastic cells that are known as spindle shaped N-cadherin+/CD45− osteoblasts (SNOs) [33]. This is the location where the previously described interaction between DTCs and osteoblastic cells via TAM receptor signaling. Osteoblasts promote dormancy maintenance in DTCs, and inhibition of their activity has been shown to promote cancer proliferation [37]. One of the ways in which SNOs promote dormancy is through Jagged-1, a Notch ligand that binds to Notch receptors on cancer cells like aggressive triple-negative breast cancer. Studies have shown that low Notch receptor expression or suppression of Jagged-1 expression results in DTC proliferation and metastasis growth [38]. Besides Notch signaling, Wnt signaling has been implicated as well in dormancy escape. Osteoblasts express Wnt5a, a non-canonical Wnt ligand which activates the receptor tyrosine kinase-like orphan receptor 2 (Ror2)/SIAH signaling axes leading to the inhibition of the Wnt/β-catenin pathway, which is believed to be a promoter of DTC proliferation and escape [39]. Restoration of the growth ability of prostate cancers was achieved by silencing Wnt5a [37].

### 3.3. Bone Marrow Adipocytes (BMAs)

**Figure 2:**
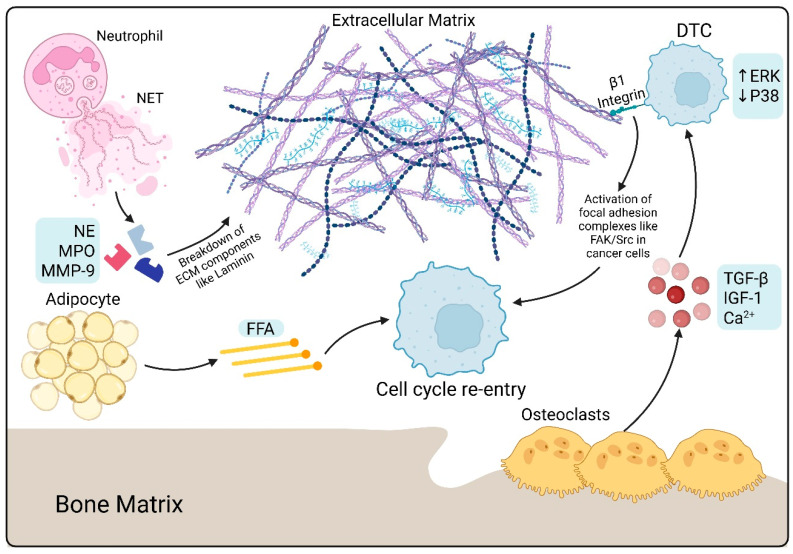
Illustration of a niche-level cascade that pushes quiescent disseminated tumor cells (DTCs) toward cell-cycle re-entry in bone. the figure depicts how NET-focused ECM remodeling, β1-integrin/FAK–Src mechanotransduction, osteoclast-derived factors, and adipocyte fuel streams cooperate to overcome dormancy and drive cell-cycle re-entry in bone. Neutrophils form NETs that concentrate proteases (NE, MPO, MMP-9), which sequentially cleave basement-membrane laminins and related ECM components; this proteolysis exposes cryptic, integrin-activating epitopes and remodels matrix architecture. The newly revealed ligands engage tumor β1-integrins, nucleating focal-adhesion signaling (FAK/Src) and shifting intracellular signaling toward an ERK^high/p38^low state that favors proliferation. In parallel, osteoclast-mediated bone resorption releases TGF-β1, IGF-1, and Ca^2+^ from the bone matrix growth-permissive cues that further bias DTCs toward reactivation and outgrowth. Bone-marrow adipocytes supply free fatty acids (FFA) to re-awakened DTCs, fueling metabolism and proliferation, while adipokine signaling adds pro- or anti-tumor pressure depending on context. Created in BioRender. Mohammad, K. (2025) https://BioRender.com/cnht3cw (accessed on 30 October 2025).

Dormant DTCs require an energy source to effectively escape dormancy and begin proliferating. Within the bone’s microenvironment, the bone marrow adipocytes (BMAs) act as the perfect source for lipids. In the presence of DTCs, phosphorylation of hormone-sensitive lipase (HSL), the rate limiting enzyme for lipolysis, and perlipin A, the gatekeeper of lipid droplets, occurs, which shifts the metabolism within BMAs from lipogenesis to lipolysis [40,41]. The free fatty acids released from the process of lipolysis are then transported to the surrounding DTCs to fuel their metabolism and proliferation [8]. In addition to fueling the metabolic processes within DTCs, BMAs release many adipokines that affect DTCs in various ways (Figure 2). Among them is leptin, an adipokine that binds to the Ob receptor on different bone components leading [9]. This adipokine has been implicated in studies on breast cancer and multiple myeloma as a major promotor of growth and metastasis as an increase in its levels correlated with a more aggressive cancer in these studies [42,43,44]. Another important adipokine that influences DTCs is adiponectin, which has major antitumor effects [45,46]. This adipokine’s secretion is found suppressed in some cancers that secrete large amounts of TNF-α like multiple myeloma, promoting DTC proliferation and growth [47]. Last but not least, resistin is another adipokine secreted by BMAs that is well documented to promote osteoclast activity, inhibit osteoblast activity, and generally change the bone microenvironment to be more favorable for DTC growth and proliferation via activating different signaling pathways like PI3K/AKT, JAK/STAT, and NFκB [48,49].

## 4. Immune System-Mediated Escape

### 4.1. Loss of Immune Surveillance

Specialized subsets of CD8+ T cells, specifically CD39 + PD-1 + CD8+ T cells, are prevalent in dormant tumor microenvironments and are essential for sustaining metastatic dormancy, especially in breast cancer models. These cells exert their influence by synthesizing effector molecules and cytokines, particularly IFN-γ and TNF-α, which induce cell cycle arrest and inhibit the proliferation of DTCs [7,50].The existence of these types of cells in both primary tumors and dormant metastases is associated with a later metastatic relapse and enhanced disease-free survival in patients, demonstrating their clinical significance [7]. Natural killer (NK) cells aggregate in tissue microenvironments where disseminated tumor cells (DTCs) remain quiescent, such as in the liver. Their presence is linked to the preservation of dormancy via IFN-γ-mediated activation of tumor cell quiescence [51,52,53]. The quantity of NK cells is critical: a substantial NK cell reservoir maintains dormancy, whereas a reduction in this population permits disseminated tumor cells to exit dormancy and develop metastases [51,52].

### 4.2. Immunosuppressive Microenvironment

Immune checkpoint inhibitors (ICI) have resulted in significant and sustained tumor regression in certain patients with metastatic cancers. The aggregation of immunosuppressive cell populations in the tumor microenvironment (TME), including myeloid-derived suppressor cells (MDSC), tumor-associated macrophages (TAM), and regulatory T cells (Treg), facilitates the emergence of immune resistance. MDSC and Treg proliferate systematically in cancer patients, suppressing T cell activation and T effector cell functionality [54].

Often in a tumor- and subset-specific manner, MDSCs use enzymatic, redox, and checkpoint pathways to inhibit CD8+ T cell activation and function. Numerous overlapping pathways that cause Cytotoxic T lymphocytes (CTLs) to malfunction or be excluded from successful responses are demonstrated by reviews and mechanistic research [7,55]. Enzymatic amino acid depletion occurs when Arginase 1 (Arg1) diminishes L-arginine levels, hence hindering TCR ζ-chain production and T cell proliferation. In addition Inducible nitric oxide synthase (iNOS) generates nitric oxide (NO) that disrupts interleukin-2 (IL-2) signaling and T cell functionality [7,55].

Reactive oxygen species/reactive nitrogen species and peroxynitrite produced by myeloid-derived suppressor cells nitrate T cell receptor and CD8-associated signaling molecules, impairing antigen-MHC recognition and triggering T cell malfunction or apoptosis [56]. The consumption of L-tryptophan by MDSC-derived indoleamine-2,3-dioxygenase (IDO) and the consequent buildup of kynurenines further enhance the suppression of T cell activation.

Upregulation of PD-L1 on MDSCs and other inhibitory ligands restricts PD-1+ T cell responses and contributes to resistance against checkpoint inhibition in certain malignancies [54,57]. MDSC-generated ROS limits CTL priming by reducing DC antigen presentation, and MDSC-derived proteases like ADAM17 can downmodulate T cell lymph-node homing receptors (like CD62L) [58,59]. Both contact-dependent and soluble mediator pathways are implicated in the direct interactions and reciprocal regulation of MDSCs and Tregs, which enhance suppression and stabilize tumor immune escape. Tumor imaging and functional studies show that when one population is disturbed, the other is frequently disturbed as well, suggesting a reciprocal dependency [60,61]. Tregs inhibit cytotoxic T cells via cytokines, receptor-mediated regulation of antigen-presenting cells, metabolic competition, and direct cytolysis; several methods are exploited by malignancies to diminish CD8+ responses. Recent syntheses highlight the redundancy of Treg pathways that maintain immunological tolerance within the tumor microenvironment [62,63]. By binding to CD80/CD86 on dendritic cells, CTLA-4 on Tregs lowers costimulation and hinders APC-dependent CD8+ T cell activation [62,63,64].

The expression of CD39/CD73 transforms extracellular ATP into adenosine, which activates A2A receptors to inhibit the effector capabilities of CD8+ T cells [60]. Inhibiting fatty acid oxidation or modifying lipid absorption (FAO inhibitors, CD36 modification) can selectively compromise the metabolic fitness of Treg/MDSC in malignancies [65,66]. In certain tumor instances, tregs can express granzyme B and perforin to cause effector T cells to undergo apoptosis [63,64]. The number of Tregs (FOXP3+, CD25high) and the expression of CTLA-4/CD39 correlate with immunosuppression and diminished responsiveness to immune checkpoint inhibitors across various tumor types [62,63]. Tumor-infiltrating myeloid-derived suppressor cells (MDSCs) can generate de novo regulatory T cells (Tregs) from naive CD4+ T cells and augment existing FOXP3+ Tregs through cell–cell interactions and soluble factors (e.g., indoleamine 2,3-dioxygenase, transforming growth factor-beta, interleukin-10) [59,60,67]. Cell–cell contacts, including β2 integrins and other adhesion molecules, are crucial for persistent connections between MDSCs and Tregs, as well as for the formation of discrete immunosuppressive niches within tumors [54,61]. Cytotoxic chemotherapies, such as gemcitabine, have been documented to decrease MDSC counts in some contexts [68].

Functional blockade, including inhibitors of Arg1, iNOS, IDO, and medicines that diminish ROS or PNT, is being assessed to alleviate enzymatic inhibition [58,59,67]. Implementing trafficking and recruitment inhibition by targeting GM CSF, VEGF, or chemokine pathways that attract MDSCs is an effective approach to restrict their accumulation [67,69].

Myeloid checkpoint modification, such as agents that influence CD11b/β2 integrin signaling or Siglec/VISTA pathways, provide novel strategies to modify MDSC activity and enhance tumor sensitivity to immune checkpoint inhibitors [54,59]. Clinical translation is hindered by cellular heterogeneity, compensatory networks, and toxicity hazards; thus, current efforts emphasize biomarker-driven combinations and temporal sequencing to optimize CD8+ reinvigoration while minimizing autoimmune [59,61,69].

The secretion of TGF-β, IL-10, PGE2, VEGF, and metalloproteinases alters the tumor microenvironment to promote immune evasion and facilitates non-immunological tumor progression [58,67]. TGF-β, IL-10, and IL-35 secreted by Tregs inhibit effector cytokine synthesis and proliferation of CD8+ T cells while reprogramming APCs to adopt tolerogenic states [62,63].

### 4.3. Inflammatory Triggers

NETs are extracellular networks composed of DNA, histones, and neutrophil proteases that exacerbate systemic inflammation, remodel the extracellular matrix through proteolytic cleavage (particularly of laminin), and subsequently reveal matrix signals that reactivate dormant tumor cells; Inhibiting NETosis or degrading NETs can offset these effects. Neutrophils release NETs during activation by infectious or sterile inflammatory stimuli; the released structure consists of chromatin (nuclear or mitochondrial DNA) adorned with histones and neutrophil granule proteins, and NETosis can occur through NOX-dependent or NOX-independent routes. NETs thus contain structural DNA/histone complexes together with enzymatic and inflammatory effectors that remain in circulation and tissues, connecting innate immunity with systemic inflammation [70,71,72].

Granular enzymes Neutrophil elastase (NE), myeloperoxidase (MPO), matrix metalloproteinases (e.g., MMP-9), and other proteases embellish the DNA scaffold and preserve catalytic function [70,71]. NETs aggregate proteases and inflammatory mediators at remote locations, disseminate cytokine and oxidant signals, and interact with coagulation and platelets to enhance systemic inflammation and thromboinflammatory conditions [70,72]. NETs release active NE and MMP-9 onto adjacent matrix fibers, thereby concentrating proteolytic activity in the area. NE and MMP-9 successively interact with laminin and other basement membrane constituents to produce cleaved fragments and expose cryptic matrix epitopes [73]. Proteolysis can reveal particular integrin-binding patterns, such as an α3β1-activating laminin epitope identified in NET-driven models, which alters cell–matrix signaling from keeping dormancy to stimulating growth [73].

NET-mediated ECM alterations modify stromal cell activity (endothelial cells, fibroblasts, osteoclast/osteoblast equilibrium in bone niches) and enhance local inflammation and vascular permeability, facilitating metastatic seeding and proliferation [70,73]. In experimental models of inflammation and metastasis, NET-associated NE and MMP-9 successively cleave laminin and similar ECM components, revealing integrin-activating epitopes (such as the α3β1-binding part) that interact with tumor cell integrins, initiating proliferation and breaking from quiescence [73]. NET proteases may potentially target tumor cells or stromal receptors to initiate pro-growth cascades; for instance, NE from NETs can interact with TLR4 on cancer cells, activating downstream mechanisms that enhance mitochondrial biogenesis and proliferation in stressed tumor cells [74].

NETs enhance local inflammation, attract supplementary myeloid cells, and elevate vascular permeability—all of which enable DTC access to growth stimulants and nutrients that surpass dormancy barriers [75]. PAD4-mediated chromatin citrullination is crucial for classical NETosis in various experimental settings. The genetic loss of PAD4 inhibits NET production, hence diminishing tumor development and metastasis in experimental models [74]. Stromal elements, including CD73, adenosine, and A2A receptor signaling, have been demonstrated to modulate NET formation. Specifically, adenosine produced by CD73, via the A2A receptor, can locally suppress NETosis, as evidenced by bone marrow stromal cell coculture models. This indicates that the stromal niche serves as a localized inhibitor of NET production [72]. Due to the antibacterial properties of NETs, systemic inhibition of NETs poses a risk of infectious consequences; methods that locally regulate NETosis (such as niche CD73 modification) or temporarily degrade detrimental NETs may enhance safety [72,76].

Inhibiting NET scaffolds (DNase) or the NETosis machinery (PAD4, GSDMD) represents unique mechanistic approaches with varying downstream consequences and safety profiles; preclinical evidence endorses both strategies, although clinical use necessitates thorough assessment [74,77]. NET targeting may enhance immunotherapy by mitigating NET-mediated immune suppression inside the tumor microenvironment; combination therapies are a prominent focus for translational research. Various methodologies demonstrate substantial preclinical evidence (genetic PAD4 knockout, DNase, repurposed pharmaceuticals like ivermectin, candidate small compounds, and stromal modulation); nonetheless, clinical efficacy and safety in human cancer patients are still being investigated in the literature [74,76,77,78,79].

## 5. Extracellular Matrix (ECM) and Mechanobiology

### 5.1. ECM Remodeling

Extracellular matrix modification, particularly collagen crosslinking and fibronectin deposition, is crucial in modulating cancer cell dormancy and bone metastases. Enhanced collagen crosslinking, frequently facilitated by enzymes such as lysyl oxidase (LOX), rigidifies the extracellular matrix (ECM) and fosters cancer cell migration, epithelial–mesenchymal transition (EMT), and metastatic proliferation. In bone, LOX-mediated collagen crosslinking modifies tissue rigidity, promoting cancer cell invasion and evasion from dormancy [80,81,82,83,84]. Certain collagen structures, particularly type III collagen-rich niches, can maintain dormancy through the activation of DDR1-STAT1 signaling, whereas disturbance of this environment induces proliferation [85,86].

Fibronectin (FN) is crucial for sustaining dormancy and facilitating metastatic development, relying upon its organization and environment. Dormant cancer cells can establish a fibrillar fibronectin matrix by integrin-mediated adhesion, facilitating survival in a quiescent mode. Reactivation and outgrowth necessitate matrix metalloproteinase (MMP)-mediated degradation of fibronectin (FN) [87,88]. Cancer cells and stromal cells in bone cooperate to deposit fibronectin and collagen, altering the niche to promote metastatic development [82,83,89]. Cancer cells in the bone microenvironment utilize fibroblasts and osteoblasts to synthesize collagen I, III, IV, and fibronectin, resulting in extracellular matrix disorder and modified bone architecture. This modification facilitates both dormancy (by supplying survival signals) and subsequent metastatic proliferation (by permitting escape from hibernation) [82,83,90].

Remodeling of the extracellular matrix via collagen crosslinking and fibronectin deposition establishes dynamic niches that can either sustain cancer cell dormancy or initiate metastatic proliferation, particularly in bone. The precise composition, architecture, and remodeling enzymes of the extracellular matrix dictate whether disseminated tumor cells stay latent or advance to overt metastasis, emphasizing these events as possible therapeutic targets for the prevention or treatment of bone metastasis.

Elevated matrix stiffness increases integrin signaling, reawakening quiescent cancer cells and facilitating bone metastasis by activating mechanotransduction pathways that foster cell proliferation, invasion, and the establishment of metastatic niches. Elevated stiffness of the (ECM) is detected by integrin receptors on cancer cells, functioning as mechanosensors. Rigid (ECM) facilitates integrin aggregation and activation, resulting in the formation of focal adhesion complexes (comprising FAK, Src, and PI3K/Akt), and initiates downstream signaling pathways including RhoA/ROCK, MAPK, and YAP/TAZ. These signals jointly modify cell shape, augment cytoskeletal contractility, improve cell motility, invasion, and survival [91,92,93,94,95,96,97,98].

Rigid (ECM) can reawaken quiescent cancer cells by enhancing integrin signaling, specifically β1 integrin, which subsequently activates focal adhesion kinase (FAK) and downstream effectors such as PI3K/Akt and β-catenin. This reactivation facilitates cell cycle re-entry, proliferation, and apoptosis resistance [93,94,95,96,98]. Elevated matrix stiffness amplifies integrin signaling, reactivating quiescent cancer cells and facilitating bone metastases through the activation of focal adhesion and subsequent mechanotransduction pathways. These alterations facilitate cancer cell proliferation, invasion, and the establishment of metastatic niches.

### 5.2. Mechanotransduction

Compliant niches, which are soft and non-stiff, include a distinctive (ECM) architecture, frequently abundant in type III collagen and fibronectin, that specifically instructs disseminated tumor cells (DTCs) to maintain a state of dormancy. Type III collagen specifically interacts with the DDR1 receptor on tumor cells, thereby triggering signaling pathways (e.g., STAT1) that promote dormancy. Alteration of this ECM composition or its arrangement can induce the reactivation and multiplication of dormant cells [85,87,88,99]. The ECM’s physical characteristics, specifically low stiffness and disordered fiber orientation, are essential for preserving the latent state [85,99,100].

Dormant niches comprise certain resident cells (e.g., osteoblasts in bone, endothelial cells in perivascular niches, mesenchymal stem cells) that engage with disseminated tumor cells (DTCs) through direct contact and secreted substances. Osteoblasts and mesenchymal stem cells release TGF-β2 and BMP7, which activate p38 signaling and induce cell cycle inhibitors such as p27, hence maintaining quiescence [30,101,102]. Endothelial cells are capable of synthesizing thrombospondin-1, hence enhancing dormancy [102].

Compliant niches frequently engage immunosuppressive cells (e.g., regulatory T cells, myeloid-derived suppressor cells, tumor-associated macrophages) that safeguard latent cells from immune eradication. These immune cells exhibit checkpoint markers (e.g., PDL1, B7-H4) and release factors that inhibit cytotoxic T cell activity, establishing an immunological-privileged milieu for dormancy [103,104]. Compliant niches sustain dormancy by ECM-mediated signaling, supportive interactions with niche cells, and immunological protection. These pathways combined establish a quiescent state, inhibiting tumor cell proliferation and facilitating prolonged cancer latency.

Mechanical stress in bone can reactivate latent cancerous cells and promote bone metastasis via multiple interrelated pathways, including osteolysis, fractures, and tumor-induced pressure. Bone resorption mediated by osteoclasts releases growth factors (e.g., TGF-β1, periostin) from the bone matrix, which can reactivate dormant tumor cells and stimulate their growth, resulting in pronounced metastases. Elevated bone resorption induced by agents such as parathyroid hormone or RANKL has been demonstrated to reawaken quiescent cancer cells in experimental settings [105,106,107,108,109]. Bone fractures or microdamage modify the local mechanical environment, enhancing bone remodeling and osteoclast activity, which subsequently may release signals that activate dormant cells [105,107,110].

The multiplication of tumors within the inflexible bone elevates intraosseous pressure, prompting osteocytes (bone mechanosensors) to release substances (e.g., CCL5, MMPs) that facilitate cancer cell invasion and proliferation [11,111]. Mechanical stress can elicit autophagic responses in cancer cells, hence augmenting their survival and spreading capabilities [112]. Osteoclasts facilitate the reactivation of dormant cells through bone resorption, whereas osteoblasts generally preserve dormancy via secreted factors (e.g., TGF-β2, GDF10) [105,107,113,114]. Cancer cells subjected to elevated stiffness or mechanical stress in the initial tumor can preserve a “mechanical memory,” increasing their likelihood of survival, reactivation from dormancy, and colonization of bone [115,116,117]. Tumor-induced bone breakdown is part of the “vicious cycle” of bone metastasis, which also produces substances that promote further bone resorption and tumor growth [106,108].

Mechanical stress in bone through osteolysis, fractures, or tumor pressure reactivates latent cancer cells mainly by modifying the bone microenvironment, enhancing osteoclast activity, and releasing growth factors that promote metastatic proliferation. Targeting these mechanobiological processes may provide novel techniques for the prevention or treatment of bone metastases.

## 6. Angiogenesis and Vascular Niches

The interplay between quiescent cancer cells and stable microvasculature in bone is pivotal to the onset and timing of bone metastasis. The stable microvasculature creates an environment that sustains cancer cell dormancy; nevertheless, alterations in the vasculature can reactivate these cells, resulting in metastatic advancement. Dormant disseminated tumor cells (DTCs) preferentially reside in perivascular niches within the bone marrow, closely associated with stable, non-sprouting microvasculature. Endothelial cells in these regions secrete factors such as thrombospondin-1, which induce and maintain cancer cell quiescence, preventing proliferation and metastatic outgrowth [10,106,118]. The SDF-1/CXCR4 axis further anchors dormant cells to these niches, reinforcing their dormant state [118,119]. Disturbance of vascular stability, by means of angiogenic sprouting or remodeling, modifies the microenvironment. Emerging neovasculature loses dormancy-inducing signals and subsequently generates molecules such as TGF-β1 and periostin, that facilitate cancer cell proliferation and the development of metastatic lesions [10,106,118].

Tumor-derived signals can potentially alter the vasculature, establishing a pro-metastatic environment that facilitates expansion [118,120]. Therapeutic approaches that preserve vascular stability or focus on the molecular mechanisms (e.g., CXCR4, E-selectin) which hold dormant cells could stop metastatic relapse by maintaining disseminated tumor cells (DTCs) in a dormant state or enhancing their responsiveness to treatment. On the other hand, therapies that unintentionally disturb vascular quiescence may activate latent cells [10,118,119].

Hypoxia-induced HIF-1α signaling and angiogenic signals collaborate to interrupt cancer cell dormancy in bone metastasis by reconfiguring the bone microenvironment, facilitating vascular remodeling, and activating gene networks that allow dormant cells to re-enter the cell cycle and spread. Hypoxia within the bone marrow niche stabilizes HIF-1α, a transcription factor that regulates the expression of genes associated with angiogenesis (e.g., VEGF), metabolic adaptability, and cell survival, thereby fostering a milieu that promotes tumor development and evasion from dormancy [121,122,123,124]. The activation of HIF-1α enhances the expression of pro-angiogenic factors (VEGF, FGF, PDGF, angiopoietins), resulting in the development of new blood vessels (angiogenic switch). This vascular remodeling enhances the supply of oxygen and nutrients, facilitating the transformation of dormant cancer cells to active proliferation and metastatic expansion [121,122,125,126,127]. Angiogenesis-related signals (e.g., E-selectin, CXCL12) within the bone vascular niche engage with HIF-1α signaling, hence enhancing mesenchymal–epithelial transition, Wnt activation, and chemokine-driven recruitment, resulting in metastatic colonization and proliferation [128,129]. Inhibiting HIF-1α or angiogenic pathways, such as VEGF inhibitors, may impede the angiogenic flip, hence preserving dormancy or diminishing metastatic proliferation in bone [126,127,130].The interaction among hypoxia, HIF-1α, and angiogenesis presents a prospective therapeutic target for the prevention or treatment of bone metastases [121,123,126,127].

## 7. Systemic and Physiological Triggers

### 7.1. Aging and Senescence

Systemic inflammation due to infection or damage promptly attracts neutrophils and monocytes to the impacted tissues. Neutrophils generate neutrophil extracellular traps (NETs), which restructure the extracellular matrix (ECM) and can stimulate dormant cells, including neoplastic cells. NETs adhere to ECM proteins, particularly laminin, and concentrate proteases (NE, MMP9) that successively cleave laminin, therefore modifying the ECM [72,131,132,133]. This reconfiguration reveals novel epitopes on ECM proteins, activating integrin signaling pathways (FAK/ERK/MLCK/YAP) in dormant cells, hence inducing their proliferation and reactivation from dormancy. In cancer models, persistent inflammation and NET development are directly associated with the reactivation of dormant metastatic cells and cancer recurrence [72,131,133,134,135].

### 7.2. Hormonal Changes

Depletion of estrogen and androgens results in a high turnover, pro-resorptive bone condition that promotes osteoclastogenesis and diminishes osteoblast activity. The cessation of hormones elevates inflammatory cytokines and modifies the regulatory factors of osteoclasts and osteoblasts, resulting in overall bone loss and niche modification that may facilitate metastatic proliferation. Estrogen insufficiency increases IL-1, IL-6, and TNF-α, which enhance RANKL expression and stimulate osteoclast development and activity [136].

Estrogen typically enhances osteoprotegerin (OPG) levels and inhibits RANKL; the absence of this hormone elevates the RANKL/OPG ratio, consequently promoting osteoclast maturation and bone resorption. Estrogen promotes osteoblast viability and Wnt/β-catenin signaling; its deficiency diminishes osteoblast activity and bone forming potential, hence exacerbating remodeling uncoupling [136,137]. Androgen deficiency (resulting from castration or androgen deprivation therapy) induces comparable elevations in osteoclast markers and bone resorption, thus heightening the risk of metastatic bone development through the same remodeling pathway [137,138].

Hormone deficiency influences particular signaling pathways and niche components that link remodeling to tumor cell activity. Various ligand-receptor interactions and intracellular pathways facilitate the transition from a dormant to a reactivation-permissive niche. TGFβ and other matrix factors (such as IGFs and fragments of MMP) are released during osteoclast-mediated resorption, and these can stimulate signaling in dormant cancer cells to encourage growth and invasiveness [138,139]. The generation of osteolytic factors (such PTHrP) and osteolysis are influenced by both genomic and nongenomic estrogen receptor signaling in tumor and bone cells; in Estrogen Receptor-positive (ER+) breast cancer models, nongenomic ER signaling might be necessary for complete osteolytic reactivation [140,141].

Osteoblasts can rewire intratumoral steroidogenesis to maintain tumor growth in the presence of androgen deprivation, and androgen deficiency can increase adhesion molecules such cadherin 11, facilitating tumor cell/osteoblast interactions [142,143]. Hormone depriving therapies (e.g., aromatase inhibitors, androgen deprivation therapy) elevate bone turnover and fracture risk, which might raise reactivation risk through accelerated resorption; in contrast, inhibiting osteoclastogenesis (OPG/RANKL inhibitors, bisphosphonates) may mitigate hormone loss-induced reactivation in preclinical models [105,136,138,139]. Castration or ovariectomy enhances bone resorption and significantly elevates the risk of bone metastases in mouse models; the suppression of osteoclastogenesis mitigated tumor reactivation in these situations [138].

### 7.3. Stress and Neural Signaling

By changing the RANKL/OPG balance that controls osteoclastogenesis, the sympathetic nervous system (SNS) modifies osteoclast differentiation both directly through adrenergic receptors on bone cells and indirectly through adrenergic signaling on stromal cells. The physiological decoy that opposes this axis is osteoprotegerin (OPG). Norepinephrine (NE), which is released by sympathetic fibers, engages β2 adrenergic receptors on osteoblasts/osteocytes, down regulates osteoblast activity, and up regulates receptor activator of nuclear factor κB ligand (RANKL), increasing RANKL-driven osteoclast differentiation [144,145]. Osteoclasts exhibit adrenergic receptors, and their resorptive activity can be influenced by catecholamines and paracrine signals from stromal cells or tumor [146,147].

The tumor stage and tumor secretome influence the outcomes of β-adrenergic stimulation; for instance, β2 adrenergic receptor activation modified the secretomes of breast cancer cells and inhibited human osteoclast differentiation in one model, whereas bone-tropic metastatic cells did not exhibit that anti-osteoclastogenic effect, indicating that cancer-derived signals modulate sympathetic nervous system effects on osteoclasts [147].

β adrenergic signaling, particularly via β2 adrenergic receptors, is the primary adrenergic pathway associated with bone resorption in metastasis, although evidence for α adrenergic involvement in this setting is scarce. Activation of β2 adrenergic receptors on stromal/osteoblastic cells induces pro-resorptive responses and paracrine substances that promote tumor colonization and osteolysis [144,148].

By upregulating RANKL and pro-inflammatory/pro-angiogenic cytokines (e.g., VEGF, IL 6), NE/epinephrine acts at β2 AR on osteoblasts/osteocytes, boosting osteoclastogenesis and facilitating tumor cell adherence and vascularization in bone [144,148,149]. Cytokines and chemokines activated by adrenergic signaling (IL-6, MIP-1α, TNFα, CXCL12/SDF-1) function as secondary mediators connecting sympathetic activity to osteoclast stimulation and tumor homing [148,150].

Activation of SNS reprograms bone immunity to promote immunosuppression and facilitates pre-metastatic niche development by increasing myeloid suppressor populations and modifying cytokine networks. Chronic β2 adrenergic receptor stimulation of osteoblasts increases IL-6 production, which promotes the proliferation and activation of bone marrow myeloid-derived suppressor cells (MDSC) through STAT3 signaling and enhances immunosuppressive effectors such as arginase 1 (Arg1) and indoleamine 2,3-dioxygenase 1 (IDO1) [151]. The sympathetic tone can impair the activity of cytotoxic T cells and NK cells in tumor environments, shifting local immunity towards tolerance and enhancing metastatic seeding and proliferation in bone [149,152].

Sympathetic innervation regulates vascular, stromal, and immunological modification that promote tumor homing and the osteolytic “vicious cycle”. Several preclinical and translational studies advocate for adrenergic inhibition and anti-resorptive or immune-targeted medicines as potential therapeutics. Sympathetic stimulation (β2 adrenergic receptors) in osteoblasts augments bone vascular density, endothelial adhesiveness, and neoangiogenesis, thereby facilitating metastatic cell arrest and colonization; in contrast, β blockers inhibited these effects in preclinical models, and retrospective clinical analyses indicate potential benefits of peri-diagnostic β blocker use in breast cancer groups [149,150].

β-blockers demonstrate mechanistic potential in inhibiting cancer progression and bone metastasis, with certain clinical data providing support, particularly in breast and melanoma cases. Nonetheless, meta-analyses and large cohort studies frequently reveal no significant impact on overall or cancer-specific survival across all cancer types, with results differing based on cancer type and patient context [153,154,155,156,157,158,159,160,161].

Activation of the sympathetic nervous system can elevate stromal SDF-1/CXCL12 and other chemotactic signals that draw tumor cells to the hematopoietic stem cell niche and bone [150]. The phenotype of cancer cells influences the effects of β-adrenergic stimulation on the niche; for instance, parental and bone-tropic breast cancer cells exhibit divergent impacts on osteoclastogenesis upon β-adrenergic receptor activation. This suggests that the timing and tumor subtype will dictate whether sympathetic nervous system modulation facilitates or impedes bone resorption [147].

Anti-resorptive agents (bisphosphonates, denosumab) continue to be the standard for mitigating skeletal morbidity by inhibiting osteoclast activity; emerging complementary strategies, informed by preclinical and retrospective clinical evidence, include targeting the sympathetic nervous system (β-blockers), the IL-6/STAT3 immunosuppressive pathway, or myeloid-derived suppressor cells (MDSC) [149,151]. Preclinical models suggest a more pronounced SNS effect when stress occurs before to seeding rather than following the establishment of metastases; clinical trial design must account for this temporal window [151].

Clinical translation must emphasize temporal and cell type specificity due to the context-dependent nature of SNS effects; the most substantiated strategy in the current literature involves the combination of agents that normalize sympathetic signaling with targeted inhibition of RANKL or IL-6/STAT3, alongside methods that restrict MDSC or osteoclast differentiation. Preclinical studies advocate for early intervention (peri-diagnostic or pre-metastatic phase) for SNS stabilization or β-blockade to diminish seeding, however established macrometastases may exhibit less sensitivity to SNS modification [147,149].

## 8. Epigenetic and Metabolic Reprogramming of Dormant Cells

### 8.1. Epigenetic Shifts

Epigenetic changes are crucial in regulating tumor cell dormancy and reactivation in bone metastasis. Dormancy is closely linked to repressive histone modifications (H3K9me3, H3K27me3), whereas escape entails histone acetylation and DNA demethylation, resulting in gene activation and metastatic proliferation. Dormancy repressive histone methylation H3K27me3 and H3K9me3: These trimethylation modifications are associated with transcriptional repression and the preservation of dormancy in disseminated tumor cells (DTCs) residing in the bone marrow. EZH2, the enzyme responsible for producing H3K27me3, enhances stemness and metastatic capability; the inhibition of EZH2 diminishes bone metastases and secondary seeding [162,163,164]. H3K4 Methylation: KMT2B and KMT2D, as H3K4 methyltransferases, contribute to the maintenance of cancer stem cell (CSC) quiescence and dormancy; their knockdown results in the escape from dormancy and heightened chemosensitivity [163,165]. Microenvironmental cues, including osteoblast-derived substances and niche interactions, sustain dormancy, in part via epigenetic control [166].

Histone Acetylation: Enhanced acetylation (e.g., through BRD4 or diminished HDAC activity) stimulates oncogene expression, facilitating reproduction and metastasis expansion. Inhibitors of HDAC can paradoxically elicit dormancy or facilitate bone metastasis, contingent upon the setting [162,167,168,169]. DNA Demethylation: The loss of DNA methylation at particular promoters results in the activation of genes that promote metastatic reactivation and proliferation [162,170,171]. The transition that occurs from dormancy to active metastasis is characterized by a change from repressive to permissive chromatin states, which involves histone modifications and alterations in DNA methylation [162,167,170]. Epigenetic alterations—specifically, the equilibrium between restrictive methylation (H3K27me3, H3K9me3) and activating acetylation/demethylation—regulate the dormancy and reactivation of bone metastatic carcinoma cells. Focusing on these alterations presents potential therapeutic opportunities; nevertheless, context-dependent effects and microenvironmental factors require meticulous consideration.

### 8.2. Metabolic Switches

Metabolic reprogramming plays a crucial role in cancer metastasis, particularly in bone tissue. Dormant metastatic cells generally rely on oxidative phosphorylation (OXPHOS), whereas reactivation and escape from dormancy are characterized by a transition to glycolysis and/or lipid metabolism [172,173,174]. Dormant metastatic cells in distant organs, such as bone, frequently display a quiescent state characterized by increased oxidative phosphorylation and decreased glycolysis. This metabolic profile facilitates long-term survival in nutrient-limited and low-proliferation environments [173,174]. In models of pancreatic and colon cancer, dormant cells that persist following oncogene ablation or glucose deprivation exhibit heightened dependence on oxidative phosphorylation (OXPHOS). Inhibition of OXPHOS has been shown to decrease recurrence. Micrometastases in bone and other locations exhibit increased OXPHOS levels relative to primary tumors, indicating a metabolic adaptation for dormancy [172].

Transition and Development: The Shift from Glycolysis to Lipid Metabolism: the shift from dormancy to active proliferation, referred to as escape, is linked to a metabolic transition towards glycolysis, commonly known as the Warburg effect, and, in certain instances, an enhancement of lipid metabolism [172,175,176,177,178]. Enhanced glycolysis promotes rapid ATP production, biosynthesis, and migration, thereby facilitating metastatic outgrowth [172,175,178,179]. Lipid metabolism, encompassing fatty acid oxidation and synthesis, is upregulated in aggressive and metastatic cells, supplying energy and precursors for proliferation [178,179,180]. The tumor microenvironment, characterized by hypoxia and stromal interactions, enhances glycolytic and lipid metabolic reprogramming during metastatic escape [172,176,178,181].

Recent studies indicate that dormant bone metastatic cells depend on oxidative phosphorylation (OXPHOS), whereas the transition to glycolysis and lipid metabolism facilitates escape and metastatic outgrowth. This metabolic plasticity characterizes metastatic progression and represents a potential therapeutic target.

The claim that escape is caused by a metabolic transition from oxidative phosphorylation (OXPHOS) to glycolysis is mostly supported by reductionist and preclinical models. Recent research shows that cancer cells can display hybrid metabolic states, using both glycolysis and OXPHOS depending on microenvironmental signals, despite the well-established Warburg effect and metabolic plasticity. Both patient-derived data and computational models show that this metabolic flexibility is context-dependent and not always responsible for escape 716. Therefore, in clinical settings, the causative relevance of this change in therapy resistance or escape is not entirely proven [182,183].

## 9. Clinical Evidence of Dormancy Escape in Bone

Delayed relapse in breast and prostate cancer bone metastases is significantly associated with their escape from dormancy, with clinical evidence suggesting this as a principal mechanism of recurrence. Delayed relapse recurrence occurring years or decades post-initial therapy is characteristic of both breast and prostate cancer, particularly in cases of bone metastases. Clinical cohorts indicate that estrogen receptor–positive (ER+) breast cancer patients frequently develop bone metastasis 8–10 years after diagnosis, with as many as 20% of early-stage patients experiencing distant metastases following extended latency, suggesting prolonged dormancy of disseminated tumor cells (DTCs) in bone [19,184,185,186].

Patients with prostate cancer may experience relapse decades following curative therapy, with the bones being the most common site of recurrence. (DTCs) can be identified in bone marrow even in limited conditions, and their presence correlates with a heightened risk of late recurrence [21,113,166,187,188,189]. Dormancy indicators, including NR2F1, decreased Ki-67, and autophagy-related proteins, are present in disseminated tumor cells (DTCs) from patients with extended disease-free periods, indicating their dormant condition [185,190,191]. The reactivation from dormancy is associated with modifications in the bone microenvironment (such as osteoblast and immunological signaling), the loss of tumor-intrinsic interferon signaling, and epigenetic changes, which might prompt disseminated tumor cells to re-start the cell cycle and develop overt metastases [113,166,187,188,192].

Clinical surveillance of circulating tumor cells (CTCs) and disseminated tumor cells (DTCs) using liquid biopsy and proliferation/apoptosis indicators (Ki-67, M30) may facilitate the prediction of late relapse risk, as dormant CTCs are more common among individuals with delayed recurrence [184,185,191,193]. Clinical data from cohorts of breast and prostate cancer strongly indicate that patterns of late relapse in bone metastases serve as indications of the escape from dormancy. The identification of dormant (DTCs) and their subsequent reactivation is fundamental to late recurrences, emphasizing the necessity for enhanced monitoring and therapeutic approaches aimed at addressing dormancy and its evasion mechanisms.

Bone frequently serves as a site for cancer recurrence after an extended latency period, owing to its distinctive milieu that facilitates tumor cell dormancy and subsequent reactivation. Bone has unique niches, including the endosteal and hematopoietic stem cell (HSC) niches, that enable (DTCs) to enter a quiescent, non-proliferative state for years or even decades. These niches possess immunological privilege and are abundant in signals (e.g., CXCL12-CXCR4, GAS6-AXL, TGF-β2, BMP-7) that promote and sustain dormancy, shielding disseminated tumor cells (DTCs) from immune surveillance and treatment [21,30,113,194,195,196].

Alterations in the bone microenvironment, including accelerated osteoclastic bone resorption, modified osteoblast activity, angiogenic sprouting, or inflammatory signals, can disturb dormancy and stimulate the proliferation of (DTCs), resulting in metastatic relapse. Osteoclast-mediated bone resorption releases growth factors that promote tumor proliferation, while diminished signals from osteoblasts that encourage dormancy may also trigger reactivation. Upon reactivation, tumor cells engage with bone cells (osteoclasts and osteoblasts) in a feedback loop: tumor cells induce bone resorption, which releases substances that further enhance tumor proliferation and bone degradation, hence continuing metastasis [65,194,197,198]. The distinctive microenvironment of bone allows cancer cells to remain quiescent for prolonged durations, escaping treatment and immune surveillance. Alterations in the microenvironment can subsequently reawaken these cells, rendering bone a common locus for late cancer recurrence.

Research on circulating tumor cells (CTCs) and (DTCs) has revealed unique molecular signatures and mechanisms that govern cancer cell dormancy, reactivation, and evasion, offering essential insights into metastasis and recurrence. Dormant DTCs and CTCs frequently display reversible cell cycle arrest (quiescence), characterized by the overexpression of cell cycle inhibitors (e.g., p27), downregulation of proliferative genes, and distinct transcriptome profiles [199,200,201,202,203].

The p38 MAPK pathway is prevalent in dormant (DTCs), whereas an ERKlow/p38high profile correlates with quiescence. Reactivation frequently entails a transition to ERKhigh/p38low signaling [202,203]. The dynamic deposition of histone H3.3 and its chaperone HIRA regulates the transition into and out of dormancy by inhibiting SKP2 and stabilizing p27, thereby establishing a direct connection between chromatin status and dormancy regulation [204].

Dormant DTCs alter their microenvironment by releasing type III collagen to maintain dormancy through DDR1-STAT1 signaling. Astrocyte-derived laminin-211 in the brain causes quiescence via dystroglycan-mediated YAP sequestration [85,205]. Dormant DTCs decrease MHC class I expression and increase immunological checkpoint molecules (e.g., PD-L1, CTLA-4), hence diminishing immune recognition and facilitating prolonged life [206,207]. The equilibrium between mTORC1 and mTORC2 activity in circulating tumor cells and bone marrow-resident cells affects dormancy vs. proliferation, with mTORC2 facilitating quiescence [208]. CTC and DTC investigations indicate that dormancy and reactivation are regulated by a complex interaction of cell-intrinsic programs, microenvironmental signals, and immune evasion strategies. Molecular markers activation, extracellular matrix remodeling, epigenetic regulation, and immunological checkpoint expression, are fundamental to these processes.

## 10. Therapeutic Opportunities

### 10.1. Targeting Osteoclast-Mediated Escape

#### 10.1.1. The Role of Denosumab in Bone Metastasis Dormancy

The bone microenvironment that promotes cancer cell reactivation and metastasis is disrupted by therapies like denosumab and bisphosphonates, which target osteoclast-mediated bone resorption. By suppressing osteoclasts, these drugs facilitate the preservation of cancer cell dormancy and diminish the likelihood of bone metastases.

Denosumab is a monoclonal antibody that attaches to RANKL, inhibiting it from interacting with RANK on osteoclast precursors. This inhibits osteoclast development, function, and viability, resulting in less bone resorption and a less conducive environment for tumor cell activation and proliferation [105,209,210,211,212,213,214]. Disruption of the mechanical or cellular niche, increased bone turnover, and localized alterations in oxygen and metabolism diminish signals that support dormancy, thereby exposing (DTCs) to growth stimuli. Osteoclast-mediated bone resorption critically releases matrix-bound growth factors, particularly TGFβ and associated cytokines, which activate pro-proliferative pathways in tumor cells, subsequently inducing reawakening [106,215,216].

The activation of osteoclasts serves as a pivotal mechanism that transforms a dormancy-permissive niche into a growth-promoting environment. Osteoclast resorption physically modifies the endosteal/vascular niche and interferes with osteoblast-derived quiescence signals, diminishing retention and dormancy signals for disseminated tumor cells (DTCs) [106]. The bone matrix retains TGFβ and various growth factors; osteoclastic resorption liberates these ligands, which subsequently activate tumor cell SMAD and other proliferative pathways [149]. RANKL, originating from stromal or osteoblastic cells or tumor-induced sources, facilitates osteoclastogenesis through the RANK→NFκB pathway and its downstream effectors; consequently, elevated RANKL levels enhance resorption and indirectly foster DTC proliferation [217]. Denosumab is a fully human monoclonal antibody that attaches to and neutralizes RANKL, inhibiting RANKL-RANK interactions and consequently obstructing osteoclast differentiation and function [105]. Denosumab inhibits osteoclast formation and bone resorption, thereby diminishing the release of matrix-bound growth factors (e.g., TGFβ) and maintaining osteoblast/stromal dormancy signals, which should mechanistically decrease the stimuli for (DTC) activation [105].

In vivo inhibition of RANKL-mediated signaling (utilizing OPG Fc, a RANKL decoy) blocked the ovariectomy-induced transformation of dormant breast cancer disseminated tumor cells into growing bone lesions, demonstrating that RANKL-driven osteoclast activity is essential for resorption-mediated reactivation in mice. Denosumab diminishes osteoclast-mediated skeletal complications and postpones skeletal-related events relative to standard antiresorptive in metastatic contexts, exhibiting effective inhibition of bone resorption in patients [33,218].

#### 10.1.2. Recent Clinical Trials Combining Denosumab with Immunotherapy in Bone Metastasis

Latest clinical research indicates growing evidence for the combination of denosumab with immune checkpoint inhibitors, with several prospective trials commenced since 2020; however, the majority concentrate on the treatment of existing metastases rather than the prevention of bone metastasis development. The BONEMET Trial was a prospective multicenter clinical study involving 16 patients with unresectable stage IV melanoma exhibiting bone metastases. The intervention in this study involved Denosumab in conjunction with dual checkpoint inhibition utilizing nivolumab and ipilimumab. The principal endpoints were extensive immune assessment (cytokines and T-cell composition) at baseline, and at weeks 4, 12, and 24. The secondary endpoints encompassed evaluations of tolerability and efficacy. The study has concluded, and the findings were released in the European Journal of Cancer in March 2024. The principal findings indicated elevated serum concentrations of CXCL-13 linked to the combination therapy, implying immune activation [219].

The DEMAIN Study (Phase II) was a prospective, single-arm maintenance trial involving patients with advanced KRAS-mutant non-small cell lung carcinoma. The intervention involved the administration of a PD-1 inhibitor every three weeks, in conjunction with denosumab 120 mg subcutaneously every four weeks. The principal endpoint was progression-free survival. The findings were shared in conference abstracts from 2023 to 2024. This study examined a maintenance therapy strategy irrespective of initial bone metastases status [220].

The CHARLI Trial (NCT03161756) was a Phase II randomized controlled study involving patients with unresectable or metastatic melanoma. The intervention comprised the addition of denosumab to standard immunotherapy with nivolumab ± ipilimumab. The principal endpoints were median progression-free survival (5-year evaluation) and grade 3–4 immune-related adverse events (2-year evaluation). The secondary endpoints encompassed overall response rate, overall survival, and toxicity profiles. The trial remains in progress, with its current status unspecified as of the latest update. This study is noteworthy as it constitutes the largest prospective trial explicitly aimed at assessing denosumab-immunotherapy combinations https://clinicaltrials.gov/study/NCT03161756 (accessed on 30 October 2025).

Retrospective studies have provided additional validation for these findings. Numerous retrospective analyses have yielded corroborative data for the combination. A multicenter study on NSCLC utilizing retrospective chart review illustrated the efficacy and safety of concurrent immunotherapy and denosumab in advanced NSCLC with bone metastases [221]. A comprehensive cohort analysis of 268 patients receiving immune checkpoint inhibitors (ICI) for bone metastases examined potential synergistic effects and identified associations between concurrent use and enhanced clinical outcomes [222]. Moreover, small institutional melanoma cohorts (*n* ≈ 29) indicated elevated response rates in patients administered pembrolizumab in conjunction with denosumab [223].

As of now, no extensive prospective randomized trials have been specifically formulated to evaluate denosumab-immunotherapy combinations for the primary prevention of bone metastasis formation. Recent studies primarily concentrate on the management of existing bone metastases, maintenance therapy for patients with advanced disease, and retrospective evaluations of concurrent treatment.

In terms of study design considerations, the majority of prospective studies have incorporated limited sample sizes, varying from 16 to 50 participants. The patient populations in the studies have been heterogeneous, featuring diverse combination strategies and dosing regimens. A further limitation is the absence of long-term follow-up data specifically concerning prevention endpoints. Future directions and ongoing research are concentrating on innovative trial designs that can rectify these deficiencies. Future trials should take into account several critical factors based on existing evidence. Initially, primary prevention studies ought to encompass randomized trials involving high-risk patients devoid of detectable bone metastases. Secondly, biomarker-driven methodologies ought to be employed to inform selection predicated on RANKL expression or immune signatures. The ideal timing of therapy should be assessed by contrasting concurrent and sequential administration strategies. Ultimately, dose optimization should be explored to ascertain the most efficacious denosumab dosing regimens in combination therapies.

#### 10.1.3. The Role of Bisphosphonates in Bone Metastasis Dormancy

Bisphosphonates (e.g., zoledronic acid) adhere to bone mineral and are absorbed by osteoclasts during bone resorption, prompting osteoclast death and suppressing their function. This also impedes the release of bone-derived growth factors that can activate latent tumor cells [210,211,224,225,226,227]. Bisphosphonates preserve dormancy in bone primarily by modifying the bone microenvironment, inhibiting osteoclast-mediated resorption, and releasing inhibitory signals from osteocytes, while certain bisphosphonates exhibit direct anti-tumor effects through mevalonate pathway inhibition, apoptosis induction, and anti-angiogenic properties. By inhibiting osteoclastic resorption, bisphosphonates diminish the release of matrix-bound growth factors, such as TGF-β and IGFs, which would typically activate dormant cells to proliferate, thus maintaining a bone microenvironment that restricts the transition from dormancy through both indirect niche alteration and cell–cell communication. These effects diminish the local growth stimuli that reactivate disseminated tumor cells and can produce osteocyte-mediated anti-tumor signals. Analyses of bone dormancy highlight that osteoclast activity and endosteal niche signals are pivotal regulators of the quiescence or proliferation of disseminated tumor cells, indicating that agents aimed at bone turnover consequently affect dormancy dynamics [228,229].

Bisphosphonates adhere to hydroxyapatite and are absorbed by osteoclasts, where nitrogen-containing compounds provoke osteoclast apoptosis and functional impairment, thereby diminishing osteolysis and the subsequent release of growth factors that activate tumor cells [229,230]. The protective effect on dormant cell outgrowth is potentially associated with this anti-resorptive action and further modulation of bone-resident cells by osteocytes, which may clarify why certain clinical benefits (such as reduced bone recurrence) are observed with bisphosphonates but are not entirely replicated by all anti-RANKL therapies [106,231].

The activation of osteocytic connexin 43 hemichannels by bisphosphonates induces ATP release, which directly inhibits the growth, migration, and invasion of breast cancer cells both in vitro and in vivo, establishing a paracrine mechanism that promotes tumor dormancy in bone [231].

N-bisphosphonates interfere with farnesyl pyrophosphate synthase (FPPS) within the mevalonate pathway, blocking the prenylation of small GTPases (Ras/Rho/Rac) essential for cytoskeletal dynamics, invasion, and survival; this results in tumor-cell apoptosis and diminished adhesion/invasion in vitro [232,233]. Moreover, bisphosphonates can augment anti-tumor immunity (γδ T-cell activation) and impede tumor-associated angiogenesis in preclinical models, consequently diminishing microenvironmental support for dormant-cell evasion and metastatic proliferation [230,234].

Preclinical studies indicate that inhibiting bone turnover before to or during initial colonization significantly decreases the formation of bone metastases, aligning with the prevention of dormant-cell proliferation rather than the elimination of established lesions [106,229]. Experimental administration of zoledronic acid during dormancy diminished the quantity of quiescent ER-positive breast cancer cells in bone and attenuated their estrogen-mediated proliferation in a murine model, thereby endorsing the potential of bisphosphonates to decrease dormant cell prevalence and modify responsiveness to activation signals [106].

#### 10.1.4. Denosumab Versus Bisphosphonates: Evidence and Insights in Cancer Cell Dormancy

Adjuvant and metastatic trials have evaluated denosumab against intravenous bisphosphonates, primarily zoledronic acid, focusing on skeletal outcomes or biomarkers instead of dormancy as primary endpoints. Principal randomized and pooled analyses demonstrated a superior delay in the onset of the first skeletal-related event and decreased radiation exposure to bone with denosumab compared to intravenous bisphosphonates [235].

In patients with bone metastases exhibiting persistent increased bone resorption markers after bisphosphonate treatment, transitioning to denosumab resulted in greater and continuing decreases in uNTX/TRAP 5b and fewer skeletal-related events compared to the continuation of intravenous bisphosphonates [217]. An exploratory analysis in multiple myeloma indicated a progression-free survival advantage for denosumab compared to zoledronic acid; however, this pertains to disease progression rather than direct dormancy metrics [236]. Preclinical evidence for dormancy exists for zoledronic acid: it diminished quiescent ER+ breast tumor cells in bone and inhibited estrogen-driven expansion in mice, with no comparable data for denosumab reported in that scenario [237]. There is insufficient information from clinical studies with primary outcomes that directly assess cancer cell dormancy or the elimination of dormant disseminated tumor cells, comparing denosumab to bisphosphonates.

### 10.2. Boosting Immune Surveillance

Preclinical and early clinical investigations indicate that the combination of Immune checkpoint inhibitors (ICIs) with osteoclast inhibitors may augment immune responses and alleviate bone degradation, potentially diminishing the likelihood of metastatic recurrence by addressing both immune evasion and the supporting bone microenvironment [238,239]. (ICIs), such as anti-PD-1 and anti-CTLA-4, reawaken T cells that have been inhibited by the tumor microenvironment, thereby reinstating anti-tumor immunity and possibly averting latent tumor cells from evading immune surveillance and triggering metastasis [238,239,240,241].

The bone microenvironment exhibits significant immunosuppression, hence constraining the efficacy of immune checkpoint inhibitors. A number of investigations on metastatic lung cancer indicate that combining immune checkpoint inhibitors with bone-modifying drugs offers no survival advantage, underscoring the necessity for additional research [238]. The efficacy of combination therapy may be dependent upon the kind of cancer, the microenvironment, and individual patient characteristics [238,239].

Vaccination techniques targeting latent tumor cells seek to either eradicate these cells or sustain them in a dormant, non-proliferative condition, thus averting their reactivation and eventual metastatic recurrence. Immunotherapies, such as cancer vaccines and adoptive cell transfer, can stimulate T cells to identify and trigger apoptosis in dormant cells, which are generally resistant to standard treatments like chemotherapy and radiation owing to their quiescent state [7,193,242,243,244,245]. Vaccines and immunotherapies enhance the immune system’s capacity to identify and eliminate dormant cells, evidenced by the association between particular T cell populations (e.g., CD39 + PD-1 + CD8+ T cells) and postponed metastatic relapse in breast cancer. Integrating immunization with approaches that diminish immunosuppressive cells (e.g., myeloid-derived suppressor cells) or checkpoint inhibitors can enhance dormancy maintenance or eliminate dormant cells, hence decreasing the chance of recurrence [193,245,246].

### 10.3. Inhibiting ECM and Angiogenesis Remodeling

Anti-angiogenic drugs and integrin inhibitors can interfere with extracellular matrix and angiogenesis remodeling, thereby averting the awakening of dormant cancer cells; nonetheless, clinical efficacy and resistance pose considerable obstacles. Anti-angiogenic drugs (e.g., VEGF inhibitors, angiostatin, endostatin) prevent the development of new blood vessels by targeting pro-angiogenic factors and signaling pathways, therefore preventing tumors and dormant cells of essential nutrients and oxygen required for reactivation and growth [247,248,249,250,251,252].

Integrin inhibitors impede cell-extracellular matrix connections by obstructing integrin-mediated signaling, essential for cell adhesion, migration, and survival. This can inhibit ECM remodeling and angiogenesis, both essential for dormant cancer cells to break dormancy and grow [253,254,255,256,257]. Tumors may acquire resistance to anti-angiogenic and integrin-targeted therapy via alternate pathways or adaptive mechanisms [248,249,250,254,258]. Despite encouraging preclinical findings, integrin inhibitors have not demonstrated consistent clinical efficacy, and anti-angiogenic drugs frequently necessitate conjunction with other therapy for optimal results [250,251,253,254,257].

### 10.4. Epigenetic and Metabolic Therapies

Epigenetic modulators, such as Histone deacetylase inhibitor (HDAC) inhibitors, and metabolic therapies, like fatty acid oxidation (FAO) inhibitors, are emerging as promising ways to sustain cancer cell dormancy or avert their reactivation in bone metastases. HDAC inhibitors can enhance the expression of genes linked with dormancy, whereas FAO inhibitors address the metabolic requirements of dormant or persister cells, potentially inhibiting metastatic progression. HDAC inhibitors, such as valproic acid, enhance the expression of dormancy-promoting genes like LIFR in breast cancer cells, particularly in hypoxic bone marrow environments, thereby sustaining a dormant state possibly inhibiting metastatic reactivation [167]. These medicines promote apoptosis, senescence, and differentiation, while inhibiting angiogenesis and metastatic gene expression; nevertheless, their actions are significantly influenced by environment and tumor type [167,259,260,261,262,263,264]. Resistance to HDAC inhibitors may arise through LIFR/STAT3 signaling, which can be addressed by co-administering JAK1 or BRD4 inhibitors [167]. Context-dependent dual effects, such as inducing dormancy in certain contexts and promoting tumor growth in others, have restricted the clinical translation of HDAC inhibitors, despite their promise in preclinical trials and certain hematologic malignancies. The efficacy of combination tactics is complicated by the reversibility and variety of epigenetic modifications, as well-documented clinical failures [264,265].

Dormant or drug-tolerant persister (DTP) cancer cells in bone metastases have enhanced fatty acid absorption and dependence on (FAO) for survival [266,267,268]. Inhibiting fatty acid oxidation (via inhibition of CPT1a, FASN, or CD36) diminishes DTP lifespan, hinders their transition from dormancy, and increases the effectiveness of chemotherapy in preclinical models [266,267,268,269,270,271,272,273]. FAO inhibition can modify the tumor microenvironment by diminishing immunosuppression and enhancing anti-tumor responses [269].

Although blocking fatty acid oxidation (FAO) is a potential tactic, preclinical and early clinical research indicate substantial obstacles. These include differential FAO reliance across stromal and tumor compartments, metabolic compensation by tumor cells (e.g., increased glucose dependency), and possible toxicities (e.g., hepatotoxicity with etomoxir). FAO inhibition may have distinct effects on the tumor microenvironment and cancer stem cells, and normal stem cell function may potentially be highlighting the necessity of careful toxicity monitoring and context-specific strategies [269,274] (Table 2).

## 11. Future Directions and Knowledge Gaps

The document “Emerging and Established Models of Bone Metastasis” states that there are presently no adequate animal models to replicate human tumor cell metastasis to the bone microenvironment and emphasizes the shortcomings of currently available models [275].

The article addresses the scarcity of in vivo bone metastasis models that accurately replicate dormancy and homing, indicating a necessity for improved models in this area [275]. Regarding Therapy Strategies: There exists ambiguity concerning the maintenance of dormancy, the eradication of dormant cells, or the induction of their activation for therapeutic targeting, as each strategy entails potential dangers and advantages [105,276]. The functions of diverse bone-resident cells (osteoblasts, osteoclasts, immune cells), extracellular matrix components, and systemic variables (aging, inflammation) in the escape from dormancy necessitate more clarification [105]. In “Gene Expression Predicts Dormant Metastatic Breast Cancer Cell Phenotype,” the authors find genes (Cfh, Gas6, Mme, Ogn) that are significantly expressed in dormant cells within bone (in mice), which correspond with disease-free survival in original tumors of human breast cancer. This indicates that these may function as predictive biomarkers [277]. The research of biomarkers associated with bone metastasis in breast cancer examines IL-1β expression, NR2F1, and others as possible indicators of bone metastasis or dormant disseminated tumor cell state [278]. A comprehensive mechanistic insight of the escape from dormancy in bone metastases is critically required. Filling these scientific deficiencies will be essential for formulating effective medicines to avert metastatic relapse and enhance outcome for patients. The article on liquid biopsy to observe tumor dormancy and early identification of disease relapse in solid tumors delineates how liquid biopsy (ctDNA, CTCs) can assist in tracking minimal residual disease and potentially identify transitions between dormancy and active disease [184].

## Figures and Tables

**Table 1:** Key Mechanisms in Dormancy Maintenance and Escape.

Maintenance			
Process	Molecular Pathway	Cellular Component	Key Mediators
p38/ERK Balance	↑p38, ↓ERK	Extracellular matrix	p38MAPK, ERK
Hypoxia	↑TGF-β2, ↑p27	-	TGF-β2, p27
Direct Interactions	TAM receptor signaling	DTC, Osteoblasts	AXL, GAS6
Paracrine	BMP signaling	Osteoblasts	BMP-7
-	Notch	SNO, DTC	Jagged-1, Notch
Wnt signaling	Ror2/SIAH	SNO	Wnt5a
Metabolic reprogramming	Phosphorylation of HSL and Perlipin A	BMA	FFA
Adipokine secretion	PI3K/AKT, JAK/STAT, NFκB	BMA	Leptin, adiponectin, resistin
Immune surveillance	-	CD8+ T-cells, NK-cells	IFN-γ, TNF-α
ECM remodeling	Integrin-mediated adhesion	ECM	Fibronectin
Mechanotransduction	STAT1	ECM	Type III collagen
Angiogenic factors	SDF-1/CXCR4	Endothelial cells	Thrombospondin-1
Epigenetic modification	Histone methylation	DTC	EZH2, H3K4 methyltransferases
**Escape**			
Direct Interactions	TAM receptor signaling	DTC, Osteoblasts	TYRO, MERTK, GAS6
Neuronal Signaling	β2-adrenergic receptor	Neurons, DTC	Norepinephrine
Bone Resorption	-	Osteoclasts	TGF-β1, IGF-1, Ca^++^
Immunosuppression	Enzymatic amino acid depletion Nitration of TCR Inducing apoptosis	MDSC, TAM, Treg	iNOS, Arg1 ROS, RNS, peroxynitrite PD-L1
ECM remodeling	Collagen crosslinking Degradation of fibronectin	Fibroblasts	LOX MMP
Hypoxia	Angiogenesis	Endothelial cells	HIF-1α, VEGF, FGF, PDGF
Aging	FAK, ERK, MLCK, YAP	Neutrophils, ECM	Laminin, MMP, NET
Hormonal depletion	Promotion of osteoclastogenesis through OPG/RANK/RANKL	Osteoclasts, osteoblasts	IL-1, IL-6, TNF-α
Epigenetic modifications	Histone acetylation and demethylation	DTC	BRD4

↑: Increased levels of; ↓: Decreased levels of.

**Table 2:** Translational Challenges and Clinical Limitations of Therapies Targeting Dormancy Escape.

Therapeutic Strategy	Mechanistic Rationale	Translational Challenges	Clinical Limitations
Osteoclast Inhibitors (Denosumab)	Blocks RANKL–RANK, reduces resorption.	Limited biomarkers; unclear timing.	ONJ risk; no direct dormancy data.
Bisphosphonates	Induce osteoclast apoptosis.	Variable patient response.	Renal toxicity; limited survival benefit.
Immune Checkpoint Inhibitors	Restore T-cell surveillance.	Dormant cells low immunogenicity.	Immune toxicities; modest bone response.
ICI + Osteoclast Inhibitors	Dual immune + niche targeting.	Sparse prospective data.	Toxicity; unclear survival benefit.
MDSC-Targeting Agents	Reverse immunosuppression.	Pathway redundancy.	Inconsistent clinical results.
NET Inhibitors (PAD4i, DNase)	Prevent ECM remodeling signals.	Infection risk; limited validation.	No approved agents.
Anti-Angiogenic Therapy	Blocks angiogenic switch.	Dormant cells near stable vessels.	Resistance; limited efficacy in bone.
Integrin/FAK Inhibitors	Block mechanotransduction.	Redundant pathways.	Off-target toxicity.
Epigenetic Modifiers	Alter chromatin states.	Broad effects; risk of activation.	Hematologic toxicity.
Metabolic Modulators	Target OXPHOS/glycolysis.	Metabolic plasticity.	Tolerance issues.
β-Blockers (SNS Modulation)	Reduce NE-driven reactivation.	Context-dependent effects.	Inconsistent clinical benefit.

## Data Availability

No new data were created or analyzed in this study. Data sharing is not applicable to this article.
